# Elevated expression of human bHLH factor ATOH7 accelerates cell cycle progression of progenitors and enhances production of avian retinal ganglion cells

**DOI:** 10.1038/s41598-018-25188-z

**Published:** 2018-05-01

**Authors:** Xiang-Mei Zhang, Takao Hashimoto, Ronald Tang, Xian-Jie Yang

**Affiliations:** 1 Stein Eye Institute, University of California, Los Angeles, CA USA; 2 Molecular Biology Institute, University of California, Los Angeles, CA USA

## Abstract

The production of vertebrate retinal projection neurons, retinal ganglion cells (RGCs), is regulated by cell-intrinsic determinants and cell-to-cell signaling events. The basic-helix-loop-helix (bHLH) protein Atoh7 is a key neurogenic transcription factor required for RGC development. Here, we investigate whether manipulating human ATOH7 expression among uncommitted progenitors can promote RGC fate specification and thus be used as a strategy to enhance RGC genesis. Using the chicken retina as a model, we show that cell autonomous expression of ATOH7 is sufficient to induce precocious RGC formation and expansion of the neurogenic territory. ATOH7 overexpression among neurogenic progenitors significantly enhances RGC production at the expense of reducing the progenitor pool. Furthermore, forced expression of ATOH7 leads to a minor increase of cone photoreceptors. We provide evidence that elevating ATOH7 levels accelerates cell cycle progression from S to M phase and promotes cell cycle exit. We also show that ATOH7-induced ectopic RGCs often exhibit aberrant axonal projection patterns and are correlated with increased cell death during the period of retinotectal connections. These results demonstrate the high potency of human ATOH7 in promoting early retinogenesis and specifying the RGC differentiation program, thus providing insight for manipulating RGC production from stem cell-derived retinal organoids.

## Introduction

Development of the vertebrate retina follows an evolutionarily conserved chronological order with retinal ganglion cells (RGCs) among the earliest born postmitotic neurons^1,2^. In birds and mammals, neurogenesis initiates in the central retina and spreads in a wave-like fashion towards the periphery. The preneurogenic progenitors occupying the peripheral retina are active in the cell cycle and express a high level of Pax6, whereas the neurogenic progenitors in the central retina express a lower level of Pax6 and progressively exit the cell cycle to adopt different neuronal fates^3,4^. The emergence of RGCs from the undifferentiated retinal epithelium coincides with the onset of early neurogenic gene expression^4–7^. The basic-helix-loop-helix (bHLH) transcription factor Atoh7 plays a critical role in RGC genesis. In the absence of Atoh7, the majority of RGCs fails to develop in mouse or zebrafish retinas^8–10^. In humans, mutations in the regulatory element or coding sequence of the ATOH7 gene underlie non-syndromic congenital retinal nonattachment and bilateral optic nerve aplasia or hypoplasia, leading to blindness at birth^11,12^. Cell lineage tracing studies have revealed that in addition to RGCs, the progeny of Atoh7-expressing progenitors also give rise to other retinal cell types with a bias towards producing early born neurons such as cone cells^13,14^. Consistent with *in vivo* lineage analyses, differentiation of mouse induced pluripotent stem cells (iPSCs) *in vitro* also shows that Atoh7-expressing retinal progenitors can generate RGCs and photoreceptor precursors^15^.

Molecular genetic analyses suggest that Atoh7 resides at the top of the regulatory hierarchy for RGC development^16–18^. Subsequent differentiation of the nascent postmitotic RGCs involves the high-mobility-group domain transcription factors Sox4 and Sox11^19^. Downstream of Sox4 and Sox11, the POU-domain transcription factors Pou4f1/Brn3a and Pou4f2/Brn3b regulate further differentiation of RGC subtypes, including their dendritic morphogenesis and central projection targets^20–23^. Recent molecular studies have shown that Pou4f2 forms a complex with the Lim-homeodomain transcription factor Islet-1 to control a large set of genes required for RGC differentiation^24,25^. Moreover, in the Atoh7 null mutant, coexpression of Pou4f2 and Islet-1 under the Atoh7 gene locus control is sufficient to complement the loss of Atoh7 activity and restore the RGC developmental program^26^.

The expression of Atoh7 is regulated by both cell-intrinsic factors and extrinsic cues. In the early neurogenic retina, Atoh7 mRNA is detected in a subset of retinal progenitors^27^. The homeobox gene Pax6, which participates in eye primordium determination and controls the pluripotency of retinal progenitors^28^, positively regulates Atoh7 transcription through its 5′ enhancers^29^. Although not fully characterized, Atoh7 expression and its activity also appear to be influenced by the bHLH neurogenic factor Ngn2/Neurog2 and the transcriptional repressor Hes1^7,30^. In addition, analyses of reporters driven by Atoh7 promoter in zebrafish and tagged Atoh7 protein in the mouse retinas suggest that Atoh7 expression is dynamically controlled in retinal progenitors and nascent RGCs^31–33^. In the vertebrate retina, disrupting cell-cell contacts or Notch signaling dramatically affects RGC development^34–36^. Furthermore, several secreted factors derived from postmitotic RGCs, including Shh, VEGF, and GDF11, assert a negative feedback regulation on RGC genesis from the remaining progenitor pool^37–41^. However, the precise molecular mechanisms of how these distinct signaling pathways converge to influence Atoh7 expression or function remain to be elucidated.

Despite the well-established requirement for Atoh7 in RGC development, it is still debatable whether Atoh7 plays a role in RGC fate determination or confers a competence state for early retinogenesis. It has been shown that mouse Atoh7 expressed from the bHLH factor Neurod1 gene locus can cause switches from amacrine and photoreceptor identities to RGC characteristics^42^, supporting that Atoh7 can promote and initiate RGC differentiation program in postmitotic neurons. However, mouse Atoh7 expression driven by a Crx promoter did not enhance RGC production, unless in the Atoh7 null background^43^, suggesting that Atoh7 alone is insufficient to dictate the RGC fate in the context of differentiating photoreceptor precursors. An attempt to express the chicken Atoh7 in dissociated retinal cultures resulted in increased photoreceptor production without significant enhancement of RGC genesis^44^. To enhance our current understanding of neurogenic mechanisms, especially the role of human ATOH7 in development and pathogenesis, we have used the developing chicken retina as an *in vivo* model system, which permits easy access during the early stages of retinogenesis, to evaluate whether human ATOH7 can impact the behavior and cell fate decisions of uncommitted retinal progenitors. Our results show that human ATOH7 is capable of inducing precocious neurogenesis among preneurogenic progenitors in the peripheral retina. Furthermore, ATOH7 is highly potent to promote the RGC fate among neurogenic progenitors in the central retina. We also provide evidence that elevating ATOH7 expression accelerates cell cycle progression and promotes cell cycle exit, leading to enhanced RGC production. These findings support a role of ATOH7 in RGC fate determination and suggest a useful strategy to manipulate RGC production in human pluripotent stem cell-derived retinal organoids.

## Results

### Induction of precocious RGC formation by ATOH7

At the onset of the chicken retinogenesis at Hamburger and Hamilton (HH) stage 15^45^, the central retina showed the initial emergence of a few Atoh7-expressing progenitors (Sup. Fig. 1A). Throughout early chicken retinogenesis, the Atoh7 mRNA was detected in only a subset of progenitor cells propagating as a central to peripheral neurogenic wave (Sup. Fig. 1A). At the peak of RGC genesis at HH stage 30 (embryonic day 6, E6), Atoh7 mRNA was expressed in a large population of progenitors occupying the ventricular zone as well as in postmitotic RGCs expressing the transcription factor Brn3a (Sup. Fig. 1B).

**Figure 1:**
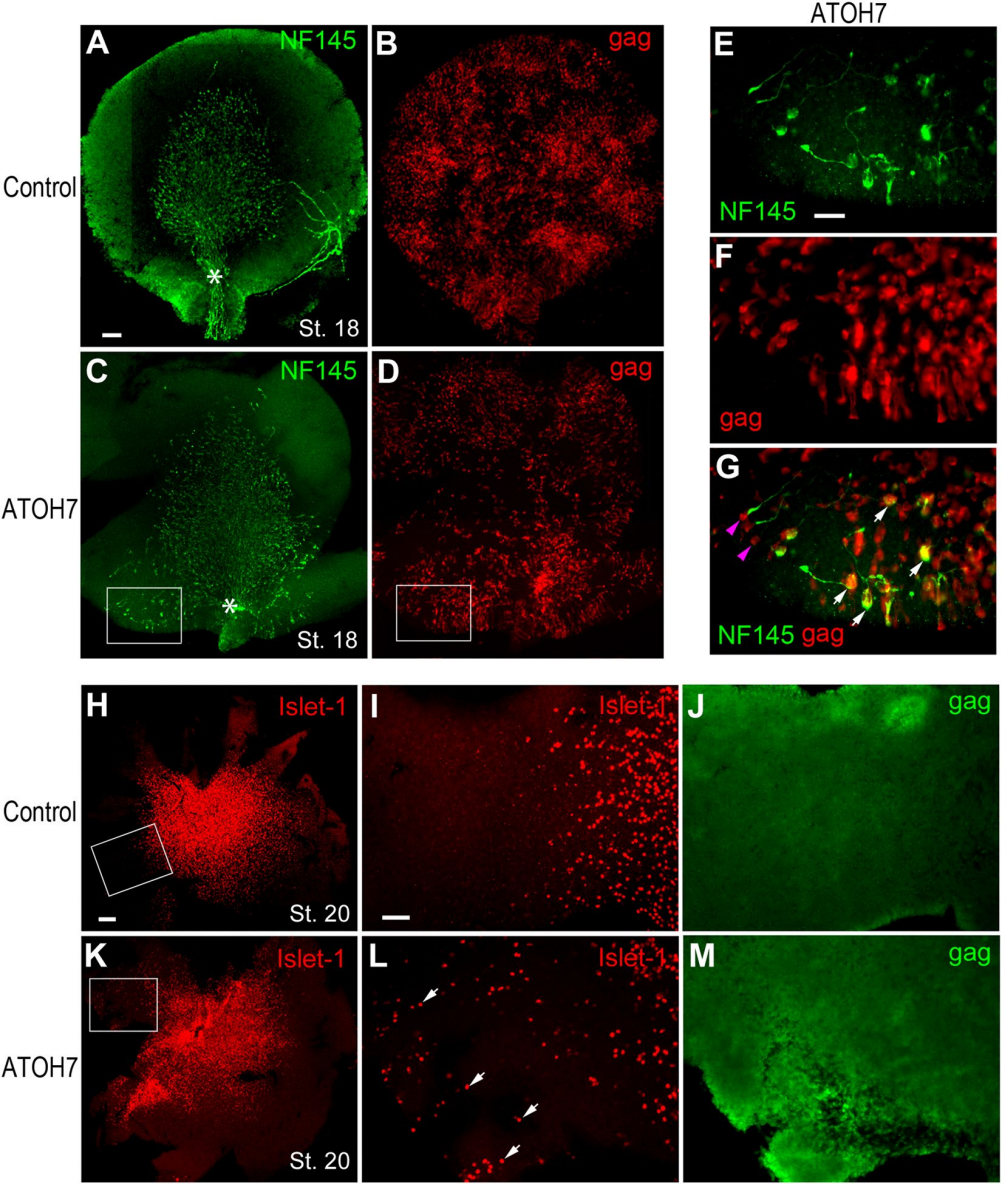
Induction of precocious retinal ganglion cell production by human ATOH7. (**A**–**D**) Immunofluorescent images of flat mount stage 18 retinas infected at stage 10 with the control virus (**A**,**B**) or ATOH7 virus (**C**,**D**) and co-labeled for NF145 (**A**,**C**) and viral gag protein (**B**,**D**). (**E**–**G**) show magnified and merged (**G**) images of the framed areas in the ATOH7 virus infected retina (**C**,**D**). White arrows in (**G**) point to ATOH7-induced ectopic RGCs co-labeled for NF145. Purple arrowheads in (**G**) indicate viral infected cells that did not express NF145. Note all NF-positive growth cones in (**E**) show abnormal projections away from the optic nerve head (white asterisks in **A**,**C**). (**H–M**) Immunofluorescent images of flat mount stage 20 retinas infected at stage 10 with the control virus (**H–J**) or ATOH7 virus (**K**–**M**) and co-labeled for Islet-1 (**H**,**I**,**K**,**L**) and viral gag protein (**J**,**M**). Framed areas in (**H**,**K**) are magnified in (**I**,**J**) and (**L**,**M**), respectively. Note that due to viral spread in the retinas (**J**,**M**), gag labeling no longer show individual infected cells. White arrows in (**L**) point to ATOH7-induced ectopic RGCs. Scale bars: A (for **A**–**D**), 20 μm; E (for **E**–**G**), 10 μm; H (for **H**,**K**), 50 μm; I (for **I**,**J**,**L**,**M**), 20 μm.

To study the neurogenic potential of Atoh7 at various stages of chicken retinogenesis, we constructed and produced an avian replication competent retrovirus encoding the human ATOH7 cDNA (RCAS.ATOH7, ATOH7 virus here in). Infection of the optic vesicle with the ATOH7 virus at HH stage 10 resulted in extensive transduction of the retina in both the preneurogenic and neurogenic regions at stage 18 as indicated by antiviral gag protein immunolabeling (Fig. 1B,D). Based on our previous characterization, neurofilaments (NFs) are among the earliest marker expressed by nascent RGCs as they exit the cell cycle and migrate away from the ventricular surface^37^. Compared to the control RCAS virus infected retinas, which showed a well-defined neurogenic wave front demarcated by NF145 (Fig. 1A), infection by RCAS.ATOH7 caused an expansion of the neurogenic area towards the peripheral retina (Fig. 1C). Immunolabeling for the transcription factor Islet-1, another RGC marker during early retinogenesis, similarly revealed ectopic neurogenesis in the peripheral retina at stage 20 (Fig. 1H–M). Interestingly, in the peripheral retina extensively infected by the ATOH7 virus (Fig. 1J,M), only a subset of viral infected cells became Islet-1-positive RGCs (Fig. 1K,L). These results show that forced expression of human ATOH7 alone is capable of inducing precocious neurogenisis in the peripheral retina, but only in a subset of preneurogenic progenitors.

### Promotion of early neuronal fates by ATOH7

**Figure 2:**
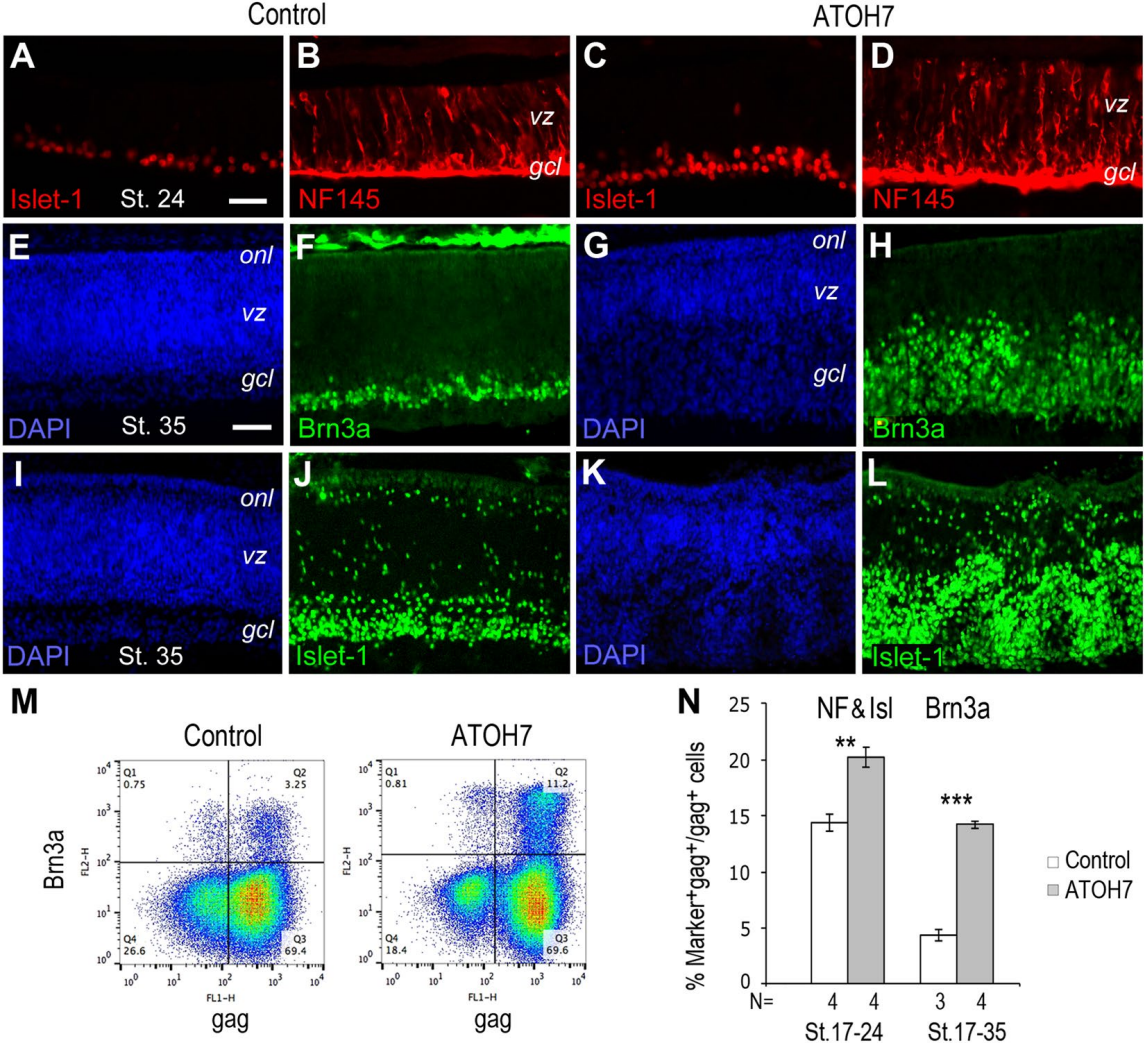
Influence of ATOH7 on retinal ganglion cell production. (**A**–**D**) Immunocytochemistry of retinas infected with the control (**A**,**B**) or ATOH7 virus (**C**,**D**) at stage 10 and labeled for Islet-1 (**A**,**C**) or NF145 (**B**,**D**) at stage 24. (**E**–**L**) Immunocytochemistry of retinas infected at stage 17 with the control (**E**,**F**,**I**,**J**) or ATOH7 virus (**G**,**H**,**K**,**L**) and labeled at stage 35 for Brn3a (**F**,**H**) or Islet-1 (**J**,**L**). The DAPI labeled panels (**E**,**G**,**I**,**K**) correspond to (**F**,**H**,**J**,**L**), respectively. Scale bars: A (for **A**–**D**), E (for **E**–**L**)10 μm. *gcl*, ganglion cell layer; *onl*, outer nuclear layer; *vz*, ventricular zone. (**M**,**N**) Flow cytometry quantification of RGC markers. (**M**) Representative flow cytometry profiles of retinas infect at stage 17 and labeled for Brn3a at stage 35. (**N**) Bar graph shows RGC marker-positive cells among gag-positive viral infected cells. Retinas infected at stage 17 were analyzed at stage 24 for NF145 and Islet-1 double positive cells, and at stage 35 for Brn3a. Numbers of independent samples analyzed and duration of viral infections are indicated below the bars. Error bars indicate SEM; **p < 0.01, ***p < 0.001.

We next examined whether human ATOH7 promoted specific neuronal cell fates during early retinogenesis. ATOH7 virus infection carried out prior to the onset of neurogenesis at stage 10 resulted in increased Islet-1 and NF145 positive cells at stage 24 (Fig. 2A–D) (Sup. Fig. 2A–D). When ATOH7 virus was delivered to the subretinal space at stage 17, as normal retinogenesis was underway, viral infection caused a significant thickening of the RGC layer occupied by Brn3a-positive postmitotic RGCs by stage 35 (Fig. 2E–H). Similar results were observed using the Islet-1 marker (Fig. 2I–L), even though its expression is not limited to RGCs at this stage.

To quantify effects of viral mediated ATOH7 expression on RGC production, we performed flow cytometry analysis using neuronal markers (Fig. 2M,N). At stage 24, compared to control virus infected cells, ATOH7 virus infection increased NF68 and Islet-1 double positive cells from 14.4 ± 0.7% to 20.2 ± 0.9% (p < 0.01) (Fig. 2N). By stage 35, ATOH7 expression resulted in 3.2-fold enhancement of Brn3a-positive RGCs from 4.4 ± 0.5% to 14.2 ± 0.3% (p < 0.001) among viral infected cells (Fig. 2M,N), whereas the RGC population remained at around 4% among the non-infected cells, similar to the control virus infected retinas.

**Figure 3:**
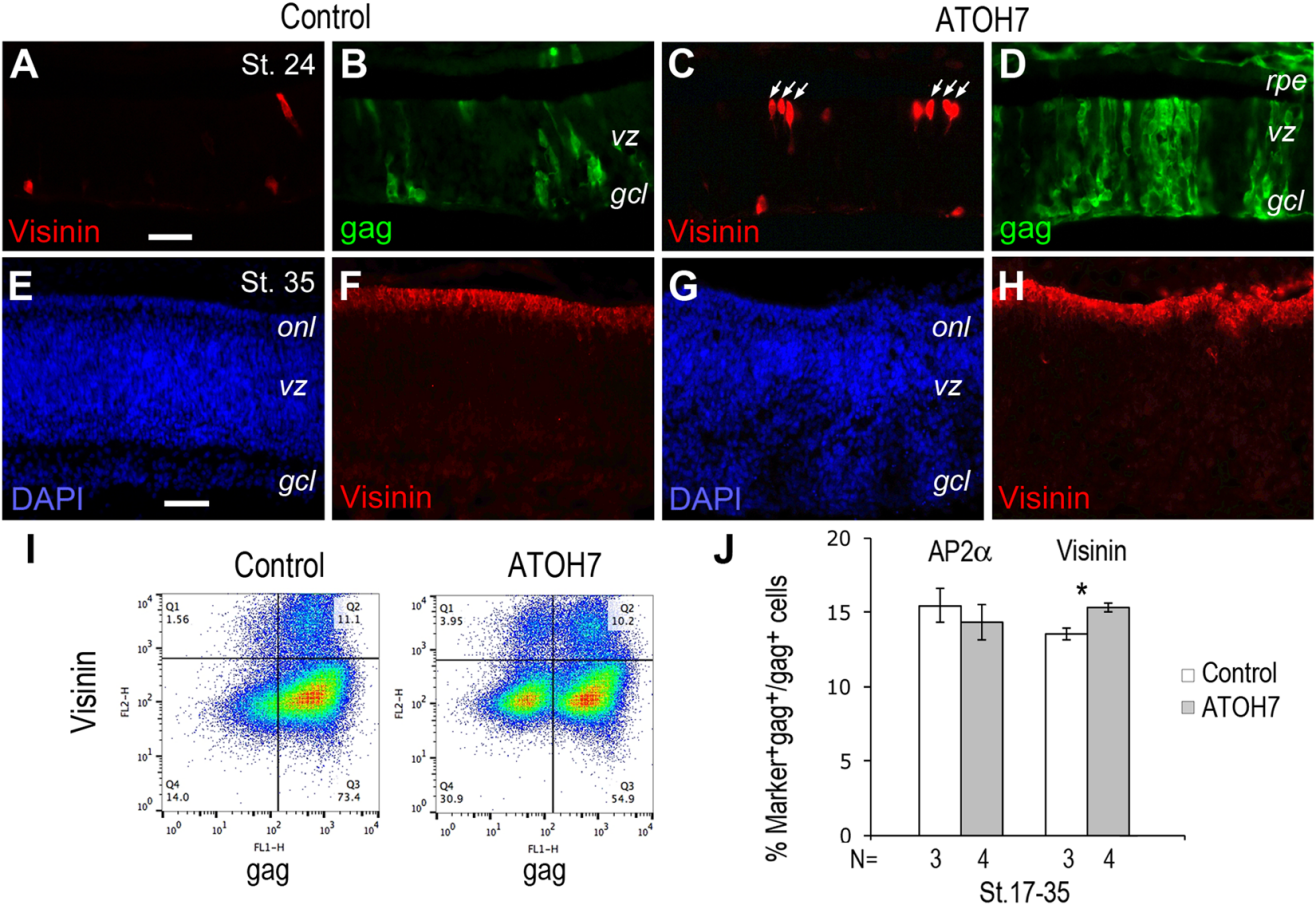
Effects of ATOH7 expression on cone photoreceptor cell production. (**A**–**H**) Immunocytochemistry of retinas infected with the control (**A**,**B**,**E**,**F**) or ATOH7 virus (**C**,**D**,**G**,**H**). Retina infected at stage 10 (**A**–**D**) or stage 17 (**E**–**H**) were labeled for visinin (**A**,**C**,**F**,**H**) and gag (**B**,**D**) at stage 24 or stage 35, respectively. Arrows in (**C**) point to visinin-positive cone cell precursors. Scale bars: A (for **A**–**D**), E (for **E**–**H**) 10 μm. *gcl*, ganglion cell layer; *onl*, outer nuclear layer; *rpe*, retinal pigment epithelium; *vz*, ventricular zone. (**I**,**J**) Flow cytometry quantification of retinal cell markers. (**I**) Representative flow cytometry profiles of Visinin at stage 35 for retinas infected at stage 17. (**J**) Quantification of amacrine marker AP2α and cone marker Visinin among gag-positive viral infected cells. Numbers of independent samples analyzed are indicated below the bars. Error bars indicate SEM. *p < 0.05.

Interestingly, ATOH7 virus infected retinas also showed an earlier onset of cone photoreceptor development as revealed by increased appearance of Visinin-positive cells near the ventricular surface (Fig. 3A–D). By stage 35, an increased number of Visinin-expressing cells were detected by immunocytochemistry in the outer retina (Fig. 3E–H) (Sup. Fig. 3). Flow cytometry quantification further confirmed that ATOH7 virus infection resulted in a minor yet statistically significant increase of Visinin-positive cone photoreceptors from 13.5 ± 0.4% to 15.3 ± 0.5% (p < 0.05) (Fig. 3I,J). Quantification of interneuron amacrine cells, as labeled by AP2α, did not show a significant change (Fig. 3J). These results demonstrate that human ATOH7 acts as a highly effective neurogenic factor and predominantly promotes the RGC fate during early chicken retinogenesis.

### Aberrant axonal projection of ATOH7-induced RGCs

The new born chicken RGCs express NF at the onset of cell body migration toward the inner retina^37^. In the control retina at stage 18, the postmitotic RGCs located in the central retina extended NF-positive processes co-expressing Tau (Sup. Fig. 4A–J), a protein predominantly located in the axons^46^. In contrast, the newly emerging RGCs in the peripheral retina only showed NF-positive cell soma without Tau (Sup. Fig. 4F–J), indicating that NF expression preceded Tau expression. Furthermore, Tau and NF double positive axons of postmitotic RGCs in the central retina became fasciculated and projected directly toward the optic nerve head (Sup. Fig. 4A–J). The ATOH7-induced precocious RGCs in the peripheral retina showed similar patterns of NF and Tau expression albeit in the expanded neurogenic territory (Sup. Fig. 4K–O). However, in contrast to the centrally projecting RGC axons found in control virus infected retinas, the emerging axons of ATOH7-induced ectopic RGCs often showed robust single growth cones misprojecting toward the periphery retina instead of the optic nerve head (Fig. 1E–G). This result suggests that certain signals orchestrating coordinated central projection may be missing or perturbed for the ectopically induced RGCs.

**Figure 4:**
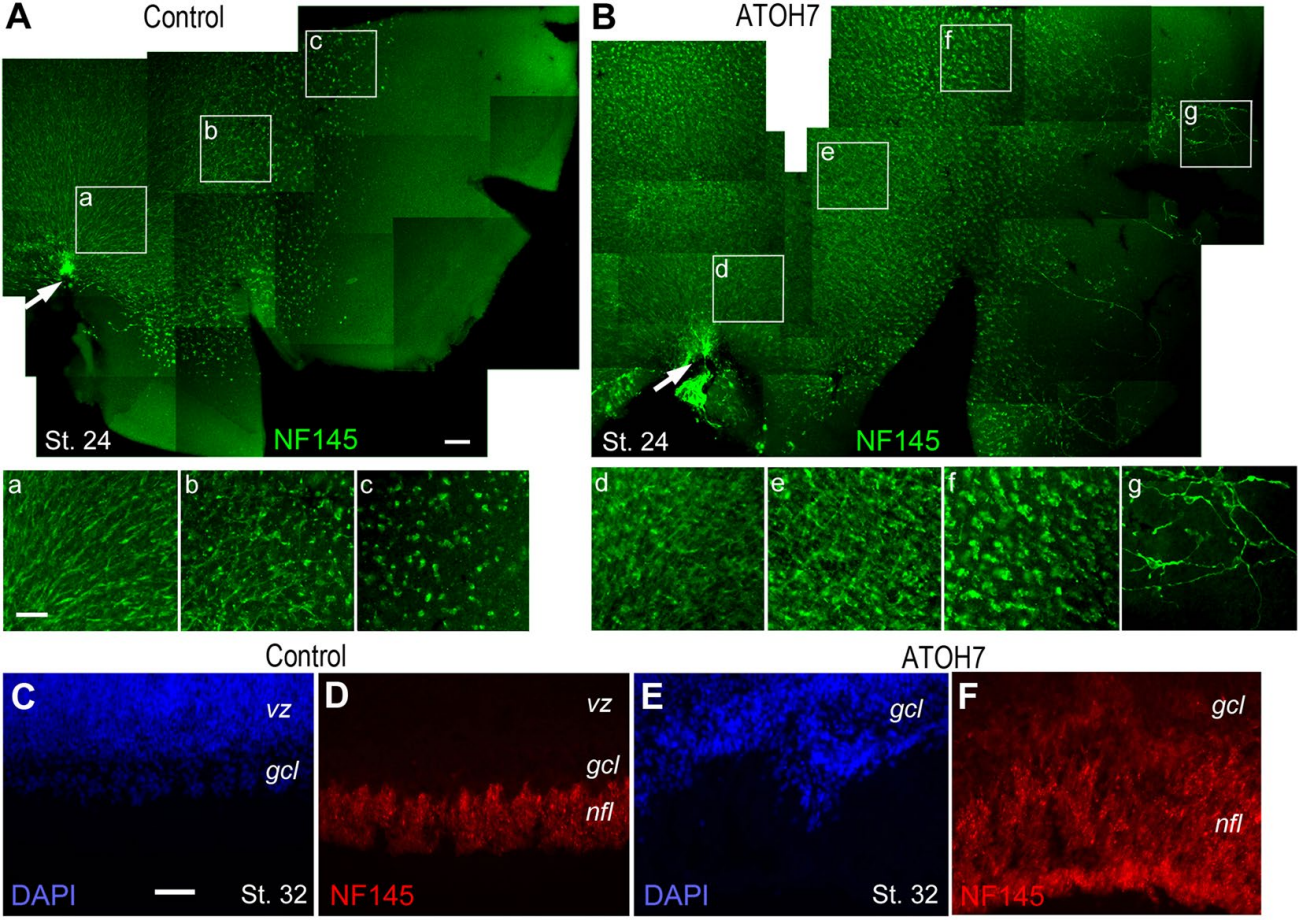
Axonal projection of human ATOH7-induced retinal ganglion cells. (**A**,**B**) Immunofluorescent images of flat mount retinas infected at stage 17 and labeled at stage 24 for NF145. The NF145-expressing retinal ganglion cells from different regions of control virus (**A**) and ATOH7 virus (**B**) infected retinas are shown at 2.5-fold magnification in (a–c) and (d–g), respectively. Arrows in (**A**) and (**B**) point to the optic nerve heads. Note the misprojected growth cones and axons of ATOH7-induced ectopic RGCs (**B**,**G**). (**C**–**F**) Immunocytochemistry of NF145 on cross sections demonstrates that retinas infected by ATOH7 virus (**E**,**F**) show increased thickness of the RGC fiber layer compared to the control virus infected retinas (**C**,**D**). *gcl*, ganglion cell layer; *nfl*, neurofiber layer; *vz*, ventricular zone. Scale bars: A (for **A**,**B**), 100 μm; a (for a–g), 50 μm; C (for **C**–**F**), 50 μm.

Since the nascent RGCs did not yet express Tau, we further examined axonal projection patterns by imaging their major growth cones, which prominently expressed NF, on retinal flat mounts at stage 24. The control retinas showed a clearly demarcated central neurogenic region containing NF-positive postmitotic RGCs (Fig. 4A). The RGCs located near the optic nerve head in the central area “a” were relatively more differentiated and showed a uniform vectorial projection pattern of growth cones towards the optic nerve head. The postmitotic RGCs in areas “b” and “c” were progressively less mature and presented shorter or no axons, respectively. In comparison, the ATOH7 virus infected retinas showed expanded neurogenic areas with precocious NF-positive neurons in the peripheral retina (Fig. 4B). The supernumerous RGCs in the more central locations “d” and “e” showed densely packed processes with more criss-cross patterns. In addition, the area “f”, which was in similar distance to the optic nerve head as the control virus infected area “c”, contained more NF-positive neurons. The abnormal axonal projections were more apparent in the peripheral retina, as shown in area “g”, which contained RGCs with elaborate wandering axons projecting to different directions. As expected, cross sections of ATOH7 virus infected retinas also displayed an increased thickness of the RGC fiber layer at stage 32 (Fig. 4C–F). This analysis reveals that precocious RGCs are prone to abnormal axonal projection and defective intraocular pathfinding.

### Impacts of ATOH7 on progenitor proliferation and cell cycle behavior

**Figure 5:**
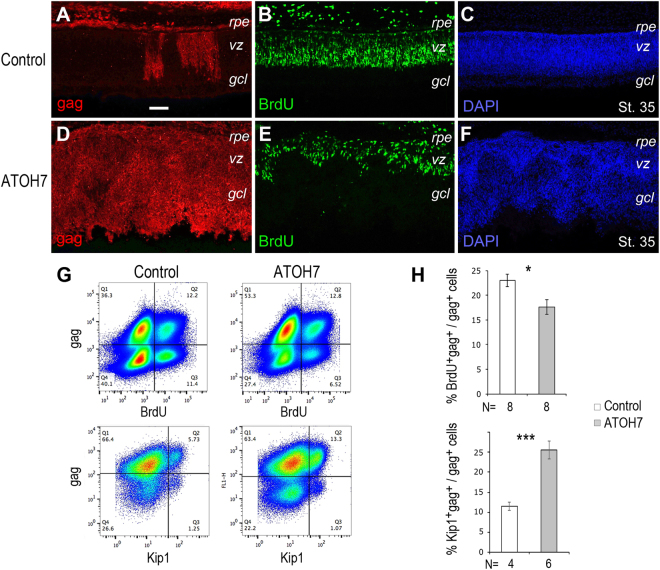
Effect of ATOH7 expression on cell proliferation. (**A**–**F**) Immunolabeled stage 35 retinal sections infected at stage 17 with the control virus (**A–C**, same section) or ATOH7 virus (**D–F**, same section). Retinas were labeled for BrdU *in vivo* for 30 minutes before fixation and analyses. Scale bar: A (for **A–F**), 20 μm. *gcl*, ganglion cell layer; *rpe*, retinal pigment epithelium; *vz*, ventricular zone. (**G**,**H**) Quantification of BrdU incorporation and p27kip1 expression. (**G**) Representative flow cytometry profiles of stage 30 retinas infected with the control virus and ATOH7 virus at stage 17. (**H**) Bar graphs show percentages of marker positive cells among gag-positive viral infected cells at stage 30. Numbers of independent samples analyzed are indicated below the bars. Error bars indicate SEM. *p < 0.05, ***p < 0.001.

We speculated that the increase of RGC production in ATOH7 virus infected retinas could impact progenitor proliferation. Immunocytochemical analysis of bromodeoxyuridine (BrdU) incorporation of stage 35 retinas revealed a marked reduction of the proliferative zone occupied by progenitors in ATOH7 virus infected retinas (Fig. 5A–F). Flow cytometry quantification of viral infected cells showed a reduction of BrdU-positive cells from 22.2 ± 1.2% in the control virus infected retinas to 17.5 ± 1.5% (p < 0.05) in ATOH7 virus infected retinas (Fig. 5G,H). Furthermore, in either control virus or ATOH7 virus infected retinas, the non-infected cell populations showed similar levels of BrdU incorporation (Sup. Fig. 5), suggesting that effects of ATOH7 expression on cell proliferation is mostly cell-autonomous. Conversely, the expression of CDK inhibitor p27kip1 was increased (Sup. Figure 2E–H). Quantification showed that p27kip1-positive cells increased more than 2-fold from 11.5 ± 1.0% to 25.6 ± 2.2% (p < 0.001) at stage 30 (Fig. 5G,H). These results suggested that a higher proportion of ATOH7 virus infected cells exited the cell cycle.

**Figure 6:**
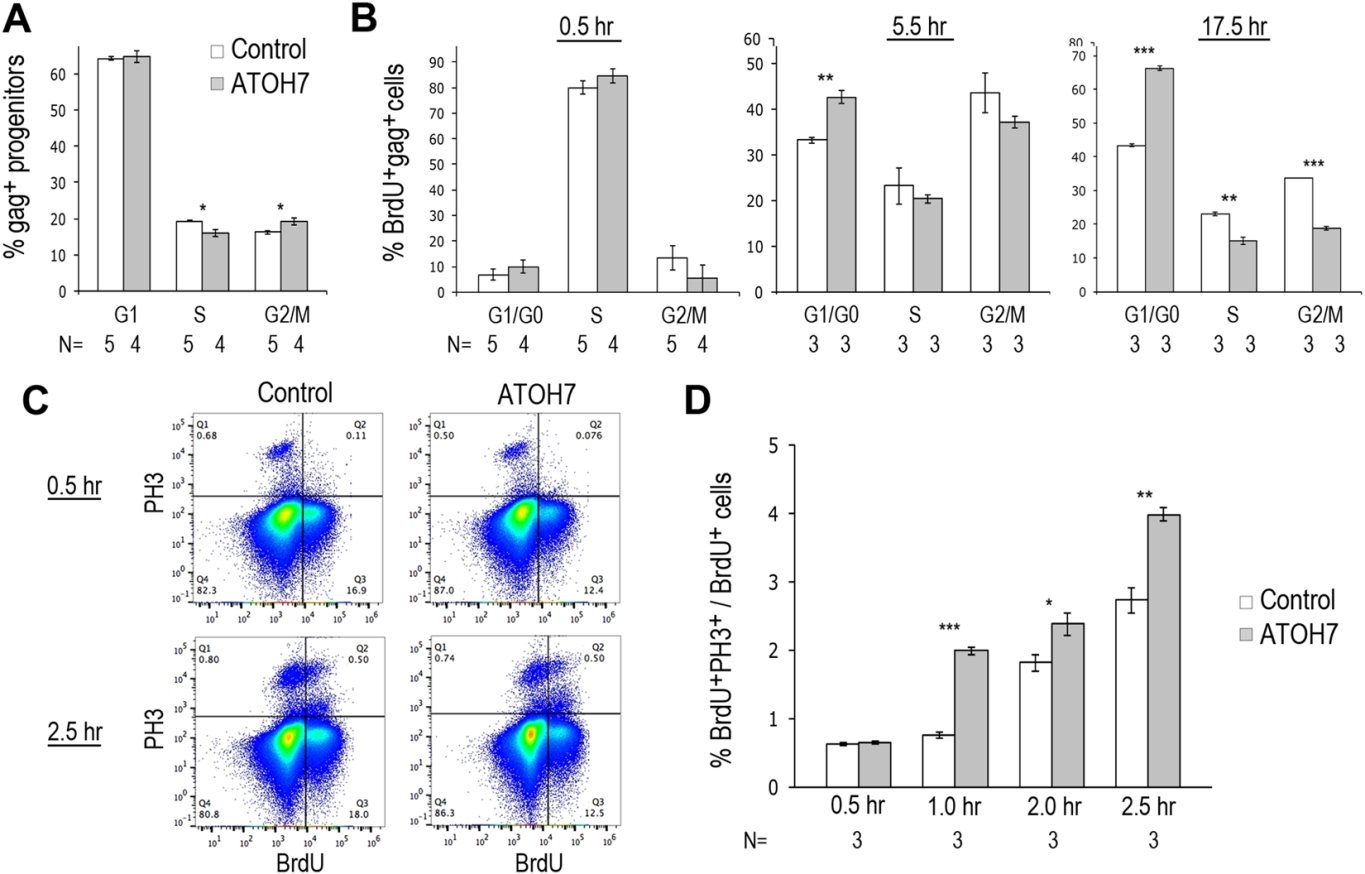
Effects of ATOH7 expression on cell cycle progression and exit. (**A**) Distribution of virally infected progenitors in different phases of the cell cycle. Retinas were infected at stage 17 and immunolabeling at stage 32 for postmitotic neuron markers, including Islet-1, NF68, Visinin, Brn3a, and AP2α−positive cells. The neuronal marker-positive cells were excluded, and the remaining progenitor populations were analyzed based on their DNA contents, with 2n cells as G1-phase cells, 4n cells as G2/M-phase cells, and those between 2n and 4n as S-phase cells. (**B**) Cell cycle progression of virus infected progenitors. Retinas were infected at stage 10, pulse labeled with BrdU at stage 30. Bar graphs show distribution of BrdU and gag double positive cells at 0.5-hour, 5.5-hour, and 17.5-hour post BrdU labeling in different phase of the cell cycle based on DNA contents. (**C**,**D**) Progression of progenitor cells from S-phase to M-phase. Retinas were infected with viruses at stage 10 and pulse labeled with BrdU at stage 30. The infection rates for the control virus and ATOH7 viruses were 75% and 54%, respectively. (**C**) Flow cytometry profiles of BrdU and PH3 double positive cells at 0.5-hour and 2.0-hour after BrdU pulse-labeling. (**D**) Bar graph shows BrdU and PH3 double positive M phase cells among BrdU-labeled cells at different time intervals. Numbers of independent samples analyzed are indicated below the bar graphs. Error bars indicate SEM. *p < 0.05; **p < 0.01; ***p < 0.001.

We next analyzed the impact of elevated ATOH7 expression on the distribution and dynamics of progenitors in different phases of the cell cycle. First, we excluded postmitotic neurons at stage 24, which included Islet-1, NF68, visinin, Brn3a, and AP2α positive cells, and analyzed the distribution of progenitors in different phases of the cell cycle based on their DNA contents (Fig. 6A) (Sup. Fig. 6A). Compared with the control virus infection, ATOH7 virus infected progenitors showed a lower percentage of S phase cells (DNA contents > 2n but < 4n, p < 0.05) and a higher proportion of G2/M phase cells (DNA content = 4n, p < 0.05). Second, we pulse-labeled retinal progenitors with BrdU, and analyzed the cell cycle distribution of viral infected cells at different time intervals after BrdU incorporation (Fig. 6B). Shortly after the BrdU pulse labeling (at 0.5 hour), control and ATOH7 virus infected cells showed similar cell cycle distributions. However, 5.5 hours after the BrdU pulse labeling, a significantly higher proportion of ATOH7 virus infected cells appeared in the G0/G1 pool, from 33.2 ± 0.6% to 42.6 ± 1.4% (p < 0.01) (Fig. 6B). By 17.5 hours post the BrdU pulse labeling, among ATOH7 virus infected cells, a significantly increased G1/G0 cohort (from 43.3 ± 0.4% to 66.2 ± 0.7%, p < 0.001) was detected. At the same time, ATOH7 virus infected cells showed a corresponding reduced S phase (from 23.1 ± 0.4% to 15.0 ± 1.0%, p < 0.01) and G2/M phase (from 33.7 ± 0.1% to 18.8 ± 0.4%, p < 0.001) distribution compared to control virus infected cells (Fig. 6B). These data indicate that despite the overall reduction of proliferating progenitor due to ATOH7 virus infection (Fig. 5), the remaining progenitors that entered the S phase showed a faster cell cycle progression. The increased G1/G0 cell distribution likely reflected increased cell cycle exit, as postmitotic neuron should have 2n DNA content.

To directly test the impact of ATOH7 expression on cell cycle progression, we further examined the time course of a cohort S-phase progenitor cells to progress through the G2 phase and enter the M phase. Cohorts of S phase progenitors were pulse labeled with BrdU and monitored for their expression of phospho-histone3 (PH3), a marker for M phase cells. Retinal samples were analyzed by flow cytometry at different time intervals to monitor the emergence of BrdU and PH3 double positive cells (Fig. 6C). We found that ATOH7 virus infected retinas showed a more rapid appearance and consistently higher percentages of BrdU and PH3 double positive cells at several short time intervals (Fig. 6D) (Sup. Fig. 6B), indicating an accelerated progression through the G2 phase and earlier M phase entry. Together, these cell cycle analyses demonstrate a strong influence of human ATOH7 on progenitor cell cycle dynamics and withdrawal.

### Influence of ATOH7 expression on RGC survival

**Figure 7:**
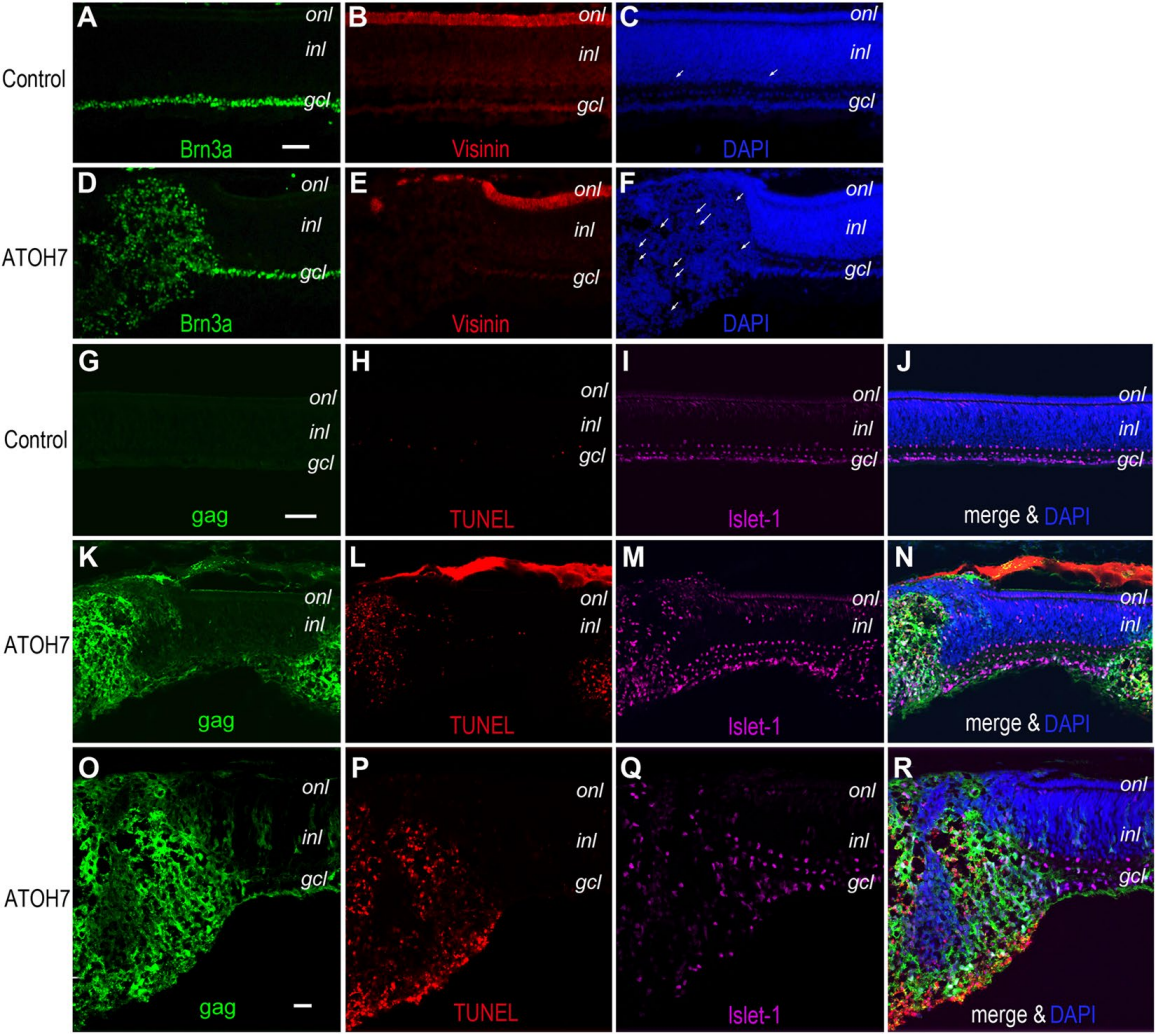
Influence of viral ATOH7 expression on neuronal survival. (**A**–**F**) Immunolabeling for neuronal markers of E9.5 retina infected at stage 17 with control virus (**A**–**C**) or ATOH7 virus (**D**–**F**). Note that ATOH7 virus infected retinas contain regions with increased numbers of RGC marker positive cells, but also disrupted lamination and extensive cell death as revealed by pyknotic nuclei (white arrows). (**G**–**R**) Co-labeling of TUNEL assay and cell markers. Non-infected control retina (**G–J**) contains few apoptotic cells at E9.5. ATOH7 virus infected retinas (**K**–**R**) show elevated TUNEL cells in gag-positive regions. Note the normal lamination and low cell death in neighboring non-infected areas. Scale bar: A (for **A**–**F**), G (for **G**–**N**) 20 μm; O (for **O**–**R**) 10 μm. *gcl*, ganglion cell layer; *inl*, inner nuclear layer; *onl*, outer nuclear layer.

The capacity of human ATOH7 to induce precocious and enhanced neurogenesis could potentially be beneficial for deriving human RGCs from stem cell sources. Therefore, we investigated the maturation and survival of ATOH7-induced RGCs during the period of retinotectal connection. Although infection of stage 17 chicken retina with ATOH7 virus led to the initial RGC overproduction, as the retina further developed, increased RGC death was detected. As shown on E9.5 retinal sections, control virus infected retinas and the regions lacking ATOH7 virus infection showed normal lamination and appeared healthy, whereas regions with augmented RGC genesis exhibited disrupted morphology and contained many pyknotic nuclei (Fig. 7A–F). To determine if the increased cell death was due to apoptosis, we performed terminal deoxynucleotidyl transferase dUTP nick-end labeling (TUNEL) assay. In the control retina, very few TUNEL positive cells were observed in the inner nuclear layer and RGC layer at E9.5 (Fig. 7G–J). In contrast, in ATOH7 virus infected retinas the regions that showed the viral gag protein expression corresponded with heavy TUNEL labeling (Fig. 7K–R).

## Discussion

In this study, we have taken advantage of the accessible embryonic chicken retina to investigate the neurogenic potential of human ATOH7 in ovo. Our results show that ectopic expression of human ATOH7 leads to precocious neurogenesis and expansion of the neurogenic territory to the peripheral retina. In the central retina undergoing active neurogenesis, elevated ATOH7 expression significantly enhances the production of postmitotic neurons, which predominantly adopt the RGC fate. Furthermore, we provide evidence that ATOH7 impacts neurogenesis among uncommitted progenitor cells by accelerating cell cycle progression and promoting cell cycle exit.

In vertebrate retinas, transition of the retinal epithelium from a preneurogenic to a neurogenic state occurs both during development and in adulthood. In adult retinas, the ciliary margin zone retains a stem or stem-like status, which is regulated to either remain quiescent or serve as a source to continuously supply progenitor cells^47,48^. During development, multiple signals regulate this transition from the pre-neurogenic state to the neurogenic state^48–50^, in which progenitors radially spanning the retinal neural epithelium are still proliferative collectively, yet are able to produce individual postmitotic neurons. While all retinal progenitors express the homeobox gene Pax6, the neurogenic progenitors downregulate Pax6 expression to a lower level^3^. In addition, the emergence of specific bHLH neurogenic factors, such as Neurog2 and Atoh7, demarcates the neurogenic zone in general^5–7^. It has been shown that FGFs are important in the onset of neurogenesis by initially inducing Atoh7^51^ and later promoting the progression of the neurogenic wave front^52^. Here, we have directly tested whether forced expression of the neurogenic factor ATOH7 among pre-neurogenic progenitors can induce a neurogenic state. Our results show that cell autonomous expression of human ATOH7 is sufficient to induce precocious neurogenesis in the peripheral retina, thus supporting a cell-intrinsic role of this factor in enabling the preneurogenic to neurogenic transition. Intriguingly, despite the wide spread viral infection, only a subset of infected progenitors enters neurogenesis in the peripheral retina. This implies that preneurogenic progenitors normally undergo additional cell intrinsic changes to become highly responsive to ATOH7 activities. This is also consistent with the observation that ATOH7 induced expansion of the neurogenic territory are primarily adjacent to the endogenous neurogenic wave front. In contrast, ATOH7 expression in the central neurogenic retina results in significantly enhanced neuronal production among viral infected progenitors, further revealing the different cell intrinsic states between the preneurogenic and the neurogenic progenitors. It is worth noting that even during the peak period of RGC genesis, only a fraction of ATOH7 virus infected cells became neurogenic in ovo. Thus, additional mechanisms, including but not limited to the known cell contact and secreted signal-mediated regulations^34–41^, likely contribute to the control of progenitor competent states.

Our results show that elevating ATOH7 expression among progenitors leads to increased cell cycle exit and neuronal production, reminiscent of Neurog2 action in the developing cortex^53,54^. In cortical progenitors, bHLH factors Hes1 and Neurog2 exhibit opposing oscillatory expression. When Neurog2 persists at high levels and Hes1 levels remains low, cortical progenitors withdraw from the cell cycle and become postmitotic neurons. Whether Atoh7 and Hes1expression oscillate in the developing retina has not been determined. In this study, the viral vector used only transduces proliferating progenitor cells and viral mediated ATOH7 expression is presumably constitutive, albeit with variable expression levels due to viral genome integration sites. By directly monitoring BrdU-labeled progenitors, we show that elevating ATOH7 expression accelerates progenitor progression through the cell cycle, especially by shortening the S phase to M phase transition. These results complement a previous report that Notch effector Hes5.3 counter reacts chicken Atoh7 accumulation to prolong the cell cycle length^55^. Despite these observations, the question persists whether Atoh7 protein are normally expressed by retinal progenitors, newly postmitotic neurons, or by both. Therefore, further investigations to determine the dynamic of endogenous Atoh7 expression during retinogenesis will be important to understand its influence on progenitor proliferation and fate decisions.

Despite the established importance of Atoh7 in RGC development, the question has remained whether its role is to provide a competent state or to specify the ganglion cell fate. Misexpressing Atoh7 in mice from a Crx promoter did not induce the RGC fate^43^, whereas replacing Neurod1 with Atoh7 altered neuronal identities from photoreceptors and amacrine cells to RGCs^42^. In contrast to the previous studies in mouse, our viral transduction approach in the embryonic chicken retina has allowed ATOH7 expression in uncommitted progenitors. Our results support the role of ATOH7 in RGC fate specification among competent progenitors during the period of RGC genesis. In addition to RGC increases, we also detected a minor but statistically significant increase of cone precursors. This is consistent with lineage tracing studies^13–15^, which show that progenies of Atoh7-expressing progenitors adopt other cell fates in addition to the RGC fate. The observed divergent effects of ATOH7 on cell fate choices may reflect the variability of ATOH7 levels among progenitor cells and dividing sibling cells. It is known that Notch signaling regulates both RGC and cone photoreceptor production and Hes1 proteins suppresses Atoh7 expression^36,40,56,57^. Thus, it is intriguing to further elucidate the dynamic changes of bHLH proteins during the neurogenic cell cycle. Important questions remain regarding the required threshold levels for ATOH7 to induce cell cycle exit, the potential interactions of ATOH7 with other transcription factors, and its effects on epigenetic programming toward a specific neuronal type.

The intraocular projection of nascent RGC axons *in vivo* follows an invariant route aiming directly towards the optic nerve head^58^. In the case of ATOH7-induced precocious RGCs, we have frequently observed aberrant axonal projection. The misprojection is particularly apparent and severe in the periphery by isolated ectopic RGCs. This phenomenon suggests that normal local and cumulative cues guiding RGC axon growth as the neurogenic wave propagates are disrupted or absent for ATOH7-induced ectopic RGCs. In addition to misrouting of axons, we also detected increased cell death among the supernumerous RGCs in ATOH7 virus infected retinas during the period of establishing retinotectal connections. This could be due to that many RGC axons fail to reach their targets, or are unable to establish stable connections within the target field. Alternatively, persistent virally driven ATOH7 expression in postmitotic RGCs may be detrimental to RGC maturation or survival.

The loss of RGCs underlies the pathology of various optic neuropathies including the major blinding disease glaucoma^59,60^. Although progresses have been made in deriving retinal neurons, the yield of human RGC production remains low in stem cell-based cultures^61–63^. It has been previously reported that chicken Atoh7 (Cath) can induce transdifferentiation of retinal pigment epithelium into photoreceptor-like cells^44^. However, viral mediated Cath misexpression only slightly enhanced RGC production^44^. In comparison, the effect we have observed for human ATOH7 to promote RGC fate in ovo is quite striking. The neurogenic potential of human ATOH7 demonstrated here may be utilized towards enhancing RGC production from stem cell-derived 3D retinal organoid cultures to facilitate disease mechanism studies and treatment development.

## Materials and Methods

### Animals

Fertilized White Leghorn chicken eggs were purchased from Charles River and incubated at 38 °C in a rotating humidified incubator. Embryos were staged according to Hamburger and Hamilton (HH stage)^45^. Operations using animals have followed regulations of UCLA Animal Research Committee.

### *In situ* hybridization

A chicken Atoh7 cDNA was generated by RT-PCR using chicken stage 24 retinal cDNA and primer pairs 5′CGGAATTCTGTATGCGTGTGAAG (XJY141) and 5′AGTAAGCTTAGCTCGTAATAAGTTC (XJY142). The resulting cDNA clone was verified by DNA sequencing. Plasmid vectors containing the chicken Atoh7 were used as DNA templates to generate digoxigenin-labeled RNA probes for *in situ* hybridization as previously described^64^.

### Viral production and injection

A full-length human ATOH7 cDNA was generated by PCR using human genomic DNA as templates and primers pairs 5′TGTGTTCATTCTGGCCCGCATCTATCAT (XJY207) and 5′AAGCGGCACATTCGTTTATTGGTCGGATTA (XJY208). The sequencing verified ATOH7 cDNA was subcloned into the avian replication competent retroviral vector RCAS(A)^65^ to generate RCAS.ATOH7. High titer RCAS.ATOH7 and control RCAS.GFP viral stocks were produced from avian DF1 cells using a transient transfection protocol^66^. Concentrated viral stocks were injected into the optic vesicle at HH stage 10 or the subretinal space at HH stage 17 as previously described^37^.

### Immunohistochemistry, TUNEL assay, and imaging

Section and whole mount immunohistochemistry was performed as described previously^38,40^. Cryosections of 14μm thickness were used for immunocytochemistry following fixation of chicken heads or eyes in 4% paraformaldehyde in PBS at 4 °C, cryoprotection with 30% sucrose in PBS, and embedding in OCT. For whole mount immunolabeling, retinas were dissected from retinal pigment epithelium and fixed in 4% paraformaldehyde in PBS. All incubations and washing steps for whole mount retinas were for more than 1 hour each. For BrdU labeling *in vivo*, 100 μg BrdU in 500 μl of PBS was dripped on top of the embryos through windowed eggshells 30 minutes before tissue harvesting. The primary antibodies used were: Neurofilament 68 (mouse, 1:400, Sigma), Neurofilament 145 (rabbit, 1:750, Chemicon), p27kip1 (mouse, 1:100, BD), Phospho-histone H3 (rabbit, 1:3000, Millipore), Pax6 (rabbit, 1:200, Chemicon), Brn3a (mouse, 1:100, Chemicon), AP2α (mouse, 1:5, DSHB), Islet-1 (mouse, clone 39.4D5, 1:10, DSHB), Visinin (mouse clone 7G4, 1:10, DSHB), Tau (chicken, 1:200, Abcam), anti-gag (mouse, clone 3C2, 1:10, DSHB; rabbit, P27, 1:125, Charles River), BrdU (mouse, 1:1, Amersham). The secondary antibodies used were conjugated to Alexa 488, 594, or 647 (1:500, Invitrogen). For nuclear staining, 4′,6-diamidino-2-phenylindole (DAPI, 1 μg/ml, Roche) was used. Fluorescent and DIC images were captured using a Nikon E800 or Olympus FluoView 1000 confocal microscopes. Cryosections were used for TUNEL assays according to manufacturer’s instructions (Roche).

### Flow cytometry, cell cycle analyses, and statistical analyses

For retinal cell marker analyses, viral infected retinas were dissected, dissociated, and subjected to flow cytometry analysis as previously described^40^. Antibody-labeled cells were filtered and collected using a tube with a 35 μm nylon mesh cap (BD falcon) before flow cytometry analyses using BD LSRII analytic flow cytometer (BD Biosciences). All experiments were performed at least twice with independent samples ranging from N = 3 to N = 8 as indicated under each bar graph shown. Each independent sample consisted of minimum one to multiple viral infected eyes depending on the markers and experiments. A minimum of 50,000 cells was passed through the flow cytometer for each individual sample (N) to obtain quantifiable data. FACS data were analyzed with FlowJo software (Tree Star, Inc.). The Student *t* test was used for pair-wise sample sets (control RCAS virus infected versus ATOH7 virus infected). Standard error of the mean (SEM) are shown in bar graphs. *P* < 0.05 is considered statistically significant.

For BrdU pulse-chase experiments, retinas were dissected first, and incubated in medium as previously described^37^ (1:1 DMEM and F12, 10 mM HEPES pH 7.0, 1% fetal calf serum, 0.2% chicken serum) containing 10 μM BrdU for 30 minutes, and then washed and exchanged into medium without BrdU, and further incubated for designated time periods at 37 °C prior to dissociation and fixation. For cell cycle analysis, cells were permeabilized by 0.1% Triton X-100 in HBSS and incubated with 1 μg/ml of 4′,6-diamidino-2-phenylindole (DAPI) for 30 minutes at room temperature. Dissociated cells were also treated with 1 N HCl for 2 min followed by washes prior to incubation with anti-BrdU antibody. Cells were analyzed by flow cytometry and FlowJo software (Tree Star, Inc.). Standard cell cycle distribution profiles based on DNA contents as measured by DAPI intensities were obtained. Different cell cycle phases were designated as follows and quantified: 2n content cells as G0 or G1-phase cells, 4n content cells as G2/M-phase cells, and those with DNA content between 2n and 4n as S-phase cells^40^.

## Electronic supplementary material

Supplemental Materirals

## References

[1] Young RW (1985). Cell differentiation in the retina of the mouse. Anat Rec.

[2] Hoon M, Okawa H, Della Santina L, Wong RO (2014). Functional architecture of the retina: development and disease. Progress in retinal and eye research.

[3] Hsieh YW, Yang XJ (2009). Dynamic Pax6 expression during the neurogenic cell cycle influences proliferation and cell fate choices of retinal progenitors. Neural development.

[4] Wan, Y. *et al*. The ciliary marginal zone of the zebrafish retina: clonal and time-lapse analysis of a continuously growing tissue. *Development*, 10.1242/dev.133314 (2016). 10.1242/dev.133314 PMC4852496 26893352

[5] Matter-Sadzinski L, Puzianowska-Kuznicka M, Hernandez J, Ballivet M, Matter JM (2005). A bHLH transcriptional network regulating the specification of retinal ganglion cells. Development.

[6] Perron M, Kanekar S, Vetter ML, Harris WA (1998). The genetic sequence of retinal development in the ciliary margin of the Xenopus eye. Developmental biology.

[7] Hufnagel RB, Le TT, Riesenberg AL, Brown NL (2010). Neurog2 controls the leading edge of neurogenesis in the mammalian retina. Developmental biology.

[8] Brown NL, Patel S, Brzezinski J, Glaser T (2001). Math5 is required for retinal ganglion cell and optic nerve formation. Development.

[9] Wang SW (2001). Requirement for math5 in the development of retinal ganglion cells. Genes Dev.

[10] Kay JN, Finger-Baier KC, Roeser T, Staub W, Baier H (2001). Retinal ganglion cell genesis requires lakritz, a Zebrafish atonal Homolog. Neuron.

[11] Ghiasvand NM (2011). Deletion of a remote enhancer near ATOH7 disrupts retinal neurogenesis, causing NCRNA disease. Nature neuroscience.

[12] Prasov L (2012). ATOH7 mutations cause autosomal recessive persistent hyperplasia of the primary vitreous. Human molecular genetics.

[13] Yang Z, Ding K, Pan L, Deng M, Gan L (2003). Math5 determines the competence state of retinal ganglion cell progenitors. Developmental biology.

[14] Brzezinski JAt, Prasov L, Glaser T (2012). Math5 defines the ganglion cell competence state in a subpopulation of retinal progenitor cells exiting the cell cycle. Developmental biology.

[15] Xie BB (2014). Differentiation of retinal ganglion cells and photoreceptor precursors from mouse induced pluripotent stem cells carrying an atoh7/math5 lineage reporter. PloS one.

[16] Mu X, Fu X, Beremand PD, Thomas TL, Klein WH (2008). Gene regulation logic in retinal ganglion cell development: Isl1 defines a critical branch distinct from but overlapping with Pou4f2. Proceedings of the National Academy of Sciences of the United States of America.

[17] Gao Z, Mao CA, Pan P, Mu X, Klein WH (2014). Transcriptome of Atoh7 retinal progenitor cells identifies new Atoh7-dependent regulatory genes for retinal ganglion cell formation. Developmental neurobiology.

[18] Mao CA (2008). Eomesodermin, a target gene of Pou4f2, is required for retinal ganglion cell and optic nerve development in the mouse. Development.

[19] Jiang Y (2013). Transcription factors SOX4 and SOX11 function redundantly to regulate the development of mouse retinal ganglion cells. The Journal of biological chemistry.

[20] Gan L (1996). POU domain factor Brn-3b is required for the development of a large set of retinal ganglion cells. Proceedings of the National Academy of Sciences of the United States of America.

[21] Xiang M, Gan L, Zhou L, Klein WH, Nathans J (1996). Targeted deletion of the mouse POU domain gene Brn-3a causes selective loss of neurons in the brainstem and trigeminal ganglion, uncoordinated limb movement, and impaired suckling. Proceedings of the National Academy of Sciences of the United States of America.

[22] Badea TC, Cahill H, Ecker J, Hattar S, Nathans J (2009). Distinct roles of transcription factors brn3a and brn3b in controlling the development, morphology, and function of retinal ganglion cells. Neuron.

[23] Sajgo S (2017). Molecular codes for cell type specification in Brn3 retinal ganglion cells. Proceedings of the National Academy of Sciences of the United States of America.

[24] Li R (2014). Isl1 and Pou4f2 form a complex to regulate target genes in developing retinal ganglion cells. PloS one.

[25] Pan L, Deng M, Xie X, Gan L (2008). ISL1 and BRN3B co-regulate the differentiation of murine retinal ganglion cells. Development.

[26] Wu F (2015). Two transcription factors, Pou4f2 and Isl1, are sufficient to specify the retinal ganglion cell fate. Proceedings of the National Academy of Sciences of the United States of America.

[27] Brown NL (1998). Math5 encodes a murine basic helix-loop-helix transcription factor expressed during early stages of retinal neurogenesis. Development.

[28] Marquardt T (2001). Pax6 is required for the multipotent state of retinal progenitor cells. Cell.

[29] Riesenberg AN (2009). Pax6 regulation of Math5 during mouse retinal neurogenesis. Genesis.

[30] Lee HY (2005). Multiple requirements for Hes 1 during early eye formation. Developmental biology.

[31] Fu X (2009). Epitope-tagging Math5 and Pou4f2: new tools to study retinal ganglion cell development in the mouse. Developmental dynamics: an official publication of the American Association of Anatomists.

[32] Boije H, Rulands S, Dudczig S, Simons BD, Harris WA (2015). The Independent Probabilistic Firing of Transcription Factors: A Paradigm for Clonal Variability in the Zebrafish Retina. Developmental cell.

[33] He J (2012). How variable clones build an invariant retina. Neuron.

[34] Austin CP, Feldman DE, Ida JA, Cepko CL (1995). Vertebrate retinal ganglion cells are selected from competent progenitors by the action of Notch. Development.

[35] Dorsky RI, Rapaport DH, Harris WA (1995). Xotch inhibits cell differentiation in the Xenopus retina. Neuron.

[36] Riesenberg AN, Liu Z, Kopan R, Brown NL (2009). Rbpj cell autonomous regulation of retinal ganglion cell and cone photoreceptor fates in the mouse retina. The Journal of neuroscience: the official journal of the Society for Neuroscience.

[37] Zhang XM, Yang XJ (2001). Regulation of retinal ganglion cell production by Sonic hedgehog. Development.

[38] Hashimoto (2006). VEGF activates divergent intracellular signaling components to regulate retinal progenitor cell proliferation and neuronal differentiation. Development.

[39] Wang Y, Dakubo GD, Thurig S, Mazerolle CJ, Wallace VA (2005). Retinal ganglion cell-derived sonic hedgehog locally controls proliferation and the timing of RGC development in the embryonic mouse retina. Development.

[40] Sakagami K, Gan L, Yang XJ (2009). Distinct effects of Hedgehog signaling on neuronal fate specification and cell cycle progression in the embryonic mouse retina. The Journal of neuroscience: the official journal of the Society for Neuroscience.

[41] Kim J (2005). GDF11 controls the timing of progenitor cell competence in developing retina. Science.

[42] Mao CA (2013). Reprogramming amacrine and photoreceptor progenitors into retinal ganglion cells by replacing Neurod1 with Atoh7. Development.

[43] Prasov L, Glaser T (2012). Pushing the envelope of retinal ganglion cell genesis: context dependent function of Math5 (Atoh7). Developmental biology.

[44] Ma W, Yan RT, Xie W, Wang SZ (2004). A role of ath5 in inducing neuroD and the photoreceptor pathway. The Journal of neuroscience: the official journal of the Society for Neuroscience.

[45] Hamburger V, Hamilton HL (1951). A series of normal stages in the development of the chick embryo. Journal of morphology.

[46] Chung PJ (2016). Tau mediates microtubule bundle architectures mimicking fascicles of microtubules found in the axon initial segment. Nature communications.

[47] Wilken MS, Reh TA (2016). Retinal regeneration in birds and mice. Current opinion in genetics & development.

[48] Moon, K. H. *et al*. Differential Expression of NF2 in Neuroepithelial Compartments Is Necessary for Mammalian Eye Development. *Developmental cell*, 10.1016/j.devcel.2017.11.011 (2017). 10.1016/j.devcel.2017.11.011 PMC5760287 29249622

[49] Fuhrmann S (2008). Wnt signaling in eye organogenesis. Organogenesis.

[50] Cabochette P (2015). YAP controls retinal stem cell DNA replication timing and genomic stability. eLife.

[51] Martinez-Morales JR (2005). Differentiation of the vertebrate retina is coordinated by an FGF signaling center. Developmental cell.

[52] McCabe KL, Gunther EC, Reh TA (1999). The development of the pattern of retinal ganglion cells in the chick retina: mechanisms that control differentiation. Development.

[53] Imayoshi, I. & Kageyama, R. Oscillatory control of bHLH factors in neural progenitors. *Trends in neurosciences*, 10.1016/j.tins.2014.07.006 (2014). 10.1016/j.tins.2014.07.006 25149265

[54] Shimojo H, Ohtsuka T, Kageyama R (2008). Oscillations in notch signaling regulate maintenance of neural progenitors. Neuron.

[55] Chiodini F (2013). A positive feedback loop between ATOH7 and a Notch effector regulates cell-cycle progression and neurogenesis in the retina. Cell reports.

[56] Yaron O, Farhy C, Marquardt T, Applebury M, Ashery-Padan R (2006). Notch1 functions to suppress cone-photoreceptor fate specification in the developing mouse retina. Development.

[57] Jadhav AP, Mason HA, Cepko CL (2006). Notch 1 inhibits photoreceptor production in the developing mammalian retina. Development.

[58] Herrera, E., Erskine, L. & Morenilla-Palao, C. Guidance of retinal axons in mammals. Seminars in cell & developmental biology, 10.1016/j.semcdb.2017.11.027 (2017). 10.1016/j.semcdb.2017.11.027 29174916

[59] Quigley HA (1993). Open-angle glaucoma. N Engl J Med.

[60] Nickells RW, Howell GR, Soto I, John SW (2012). Under pressure: cellular and molecular responses during glaucoma, a common neurodegeneration with axonopathy. Annual review of neuroscience.

[61] Sluch VM (2015). Differentiation of human ESCs to retinal ganglion cells using a CRISPR engineered reporter cell line. Scientific reports.

[62] Ohlemacher SK (2016). Stepwise Differentiation of Retinal Ganglion Cells from Human Pluripotent Stem Cells Enables Analysis of Glaucomatous Neurodegeneration. Stem cells.

[63] Teotia P (2017). Generation of Functional Human Retinal Ganglion Cells with Target Specificity from Pluripotent Stem Cells by Chemically Defined Recapitulation of Developmental Mechanism. Stem cells.

[64] Yang X-J, Cepko CL (1996). Flk-1, a receptor for vascular endothelial growth factor (VEGF), is expressed by retinal progenitor cells. The Journal of neuroscience: the official journal of the Society for Neuroscience.

[65] Hughes SH, Greenhouse JJ, Petropoulos CJ, Sutrave P (1987). Adaptor plasmids simplify the insertion of foreign DNA into helper-independent retroviral vectors. Journal of virology.

[66] Yang X-J (2002). Retrovirus-mediated gene expression during chick visual system development. Methods.

