# Supplementary material for: Elevated expression of human bHLH factor ATOH7 accelerates cell cycle progression of progenitors and enhances production of avian retinal ganglion cells
Source: Sci Rep. 2018 May 1;8:6823. doi: 10.1038/s41598-018-25188-z (PMC5931526; doi:10.1038/s41598-018-25188-z)
Supplement: Supplementary file 1 — Supplemental Materirals [file 41598_2018_25188_MOESM1_ESM.pdf]

Elevated expression of human bHLH factor ATOH7 accelerates cell cycle progression of progenitors and enhances production of avian retinal ganglion cells

Xiang-Mei Zhang[1], Takao Hashimoto[1], Ronald Tang[1], and Xian-Jie Yang[1,2]

[1]Stein Eye Institute, [2]Molecular Biology Institute, University of California, Los Angeles, CA, USA

Supplemental Figures and Legends

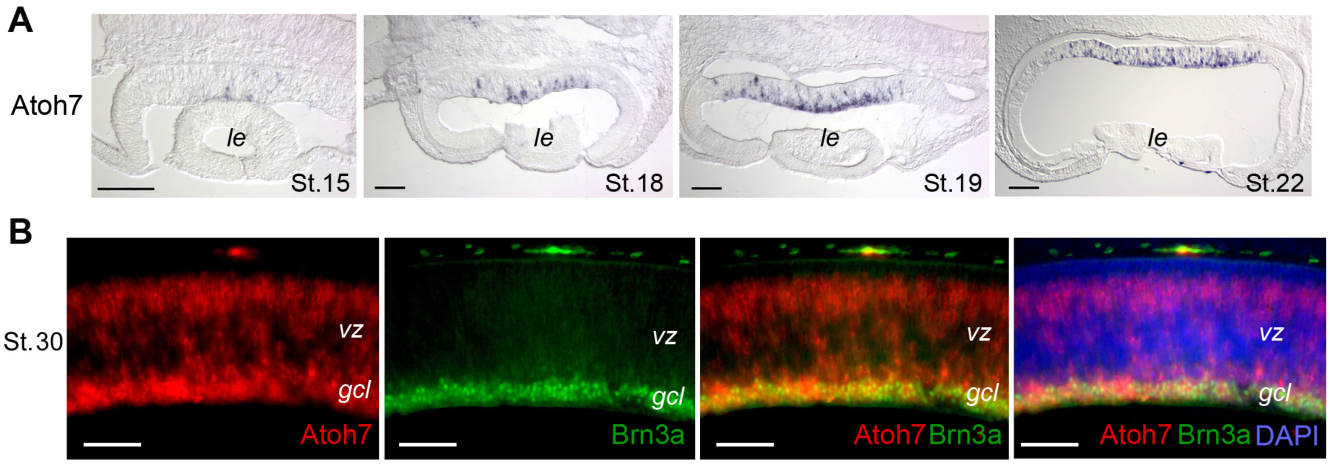

Sup. Figure 1. Expression of Atoh7 mRNA in the developing chicken retina.

(A) Chicken Atoh7 mRNA expression as detected by in situ hybridization. Few Atoh7 expressing cells were detected in the central retina at HH stage 15, and the expression spreads from the center to the periphery during early retinogenesis.
(B) Double labeling for Atoh7 mRNA by fluorescent in situ hybridization (red) and RGC marker Brn3a by immunocytochemistry (green) at stage 30. Chicken Atoh7 mRNA is expressed in a subset of progenitor cells and persists in postmitotic RGCs at stage 30 in the chicken retina.
Scale bars: A, all 100 µm; B, all 50µm. *gcl,* ganglion cell layer; *le*, lens; *vz,* ventricular zone.

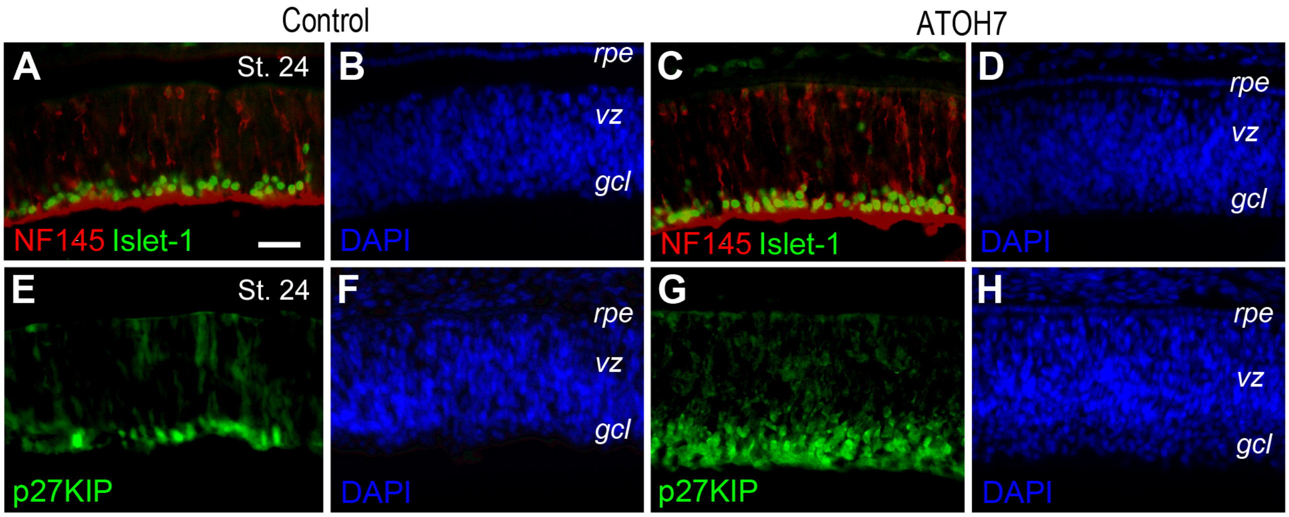

Sup. Figure 2. Influence of ATOH7 expression on RGC genesis and progenitor cell cycle exit.

Immunolabeling of stage 24 retinas infected at stage 10 are shown. Compared to the controls (A, B, E, F), ATOH7 virus infected retinas (C, D, G, H) show increased labeling for RGC markers NF145 and Islet-1 (A-D) and increased p27KIP1-positive postmitotic neurons in the RGC layer (E-H).
Scale bar: A for all, 20 µm. *gcl,* ganglion cell layer; *rpe*, retinal pigment epithelium; *vz,* ventricular zone.

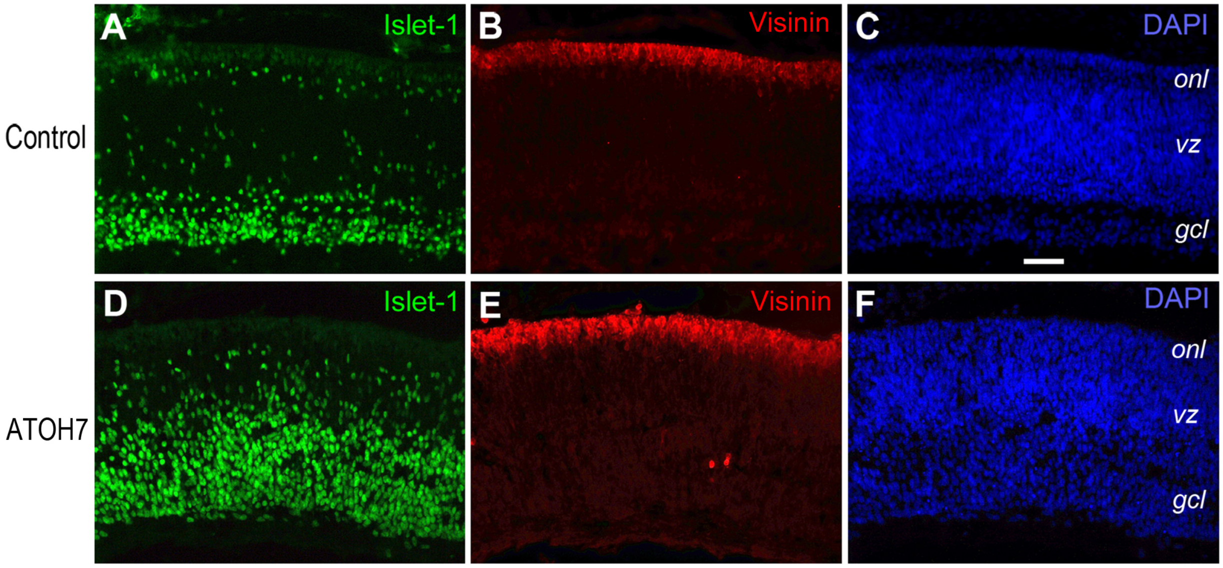

Sup. Figure 3. Influence of forced ATOH7 expression on cone photoreceptor production.

Immunolabeling of stage 35 retinas infected at stage 17 are shown. Compared to the control retinal section (A, B, C), ATOH7 virus infected retina (D, E, F) show expanded Islet-1 expressing zone (D). Consistent with the FACS quantification, in the same viral infected region, increased Visinin-positive cone cell precursors can be detected (E).
Scale bar: C for all, 10 µm. *gcl,* ganglion cell layer; *onl,* outer nuclear layer; *vz,* ventricular zone.

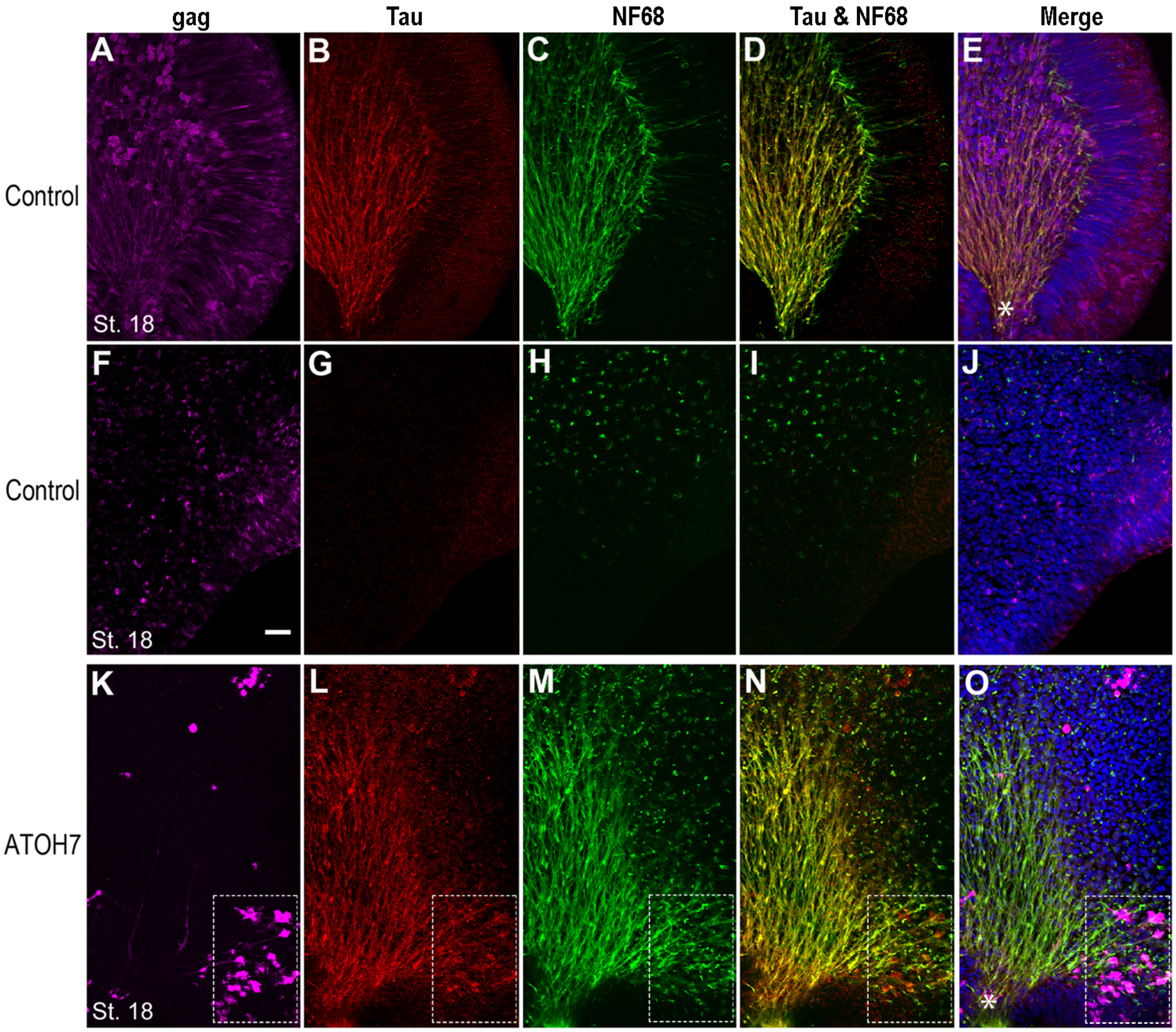

Sup. Figure 4. Expression of Tau and neurofilaments by RGCs.

Co-immunolabeling images of stage 18 flat-mount retinas infected at stage 10 are shown. (A-J) show control virus infected central (A-E) and peripheral (F-J) retina. Note that Tau protein is only detected in the more centrally located RGCs, which become postmitotic earlier thus more mature, but not expressed by newly emergent RGCs, which only express NF, in the peripheral retina. Thus, Tau is not suitable as an early axon marker for nascent RGCs. (K-O) show RCAS. ATOH7 virus infected retina with precocious emergence of RGCs (dotted frames), which show Tau expression as they are relatively more differentiated.

The white asterisks in (E) and (O) indicate the optic nerve head. Scale bar: F for all, 20 µm.

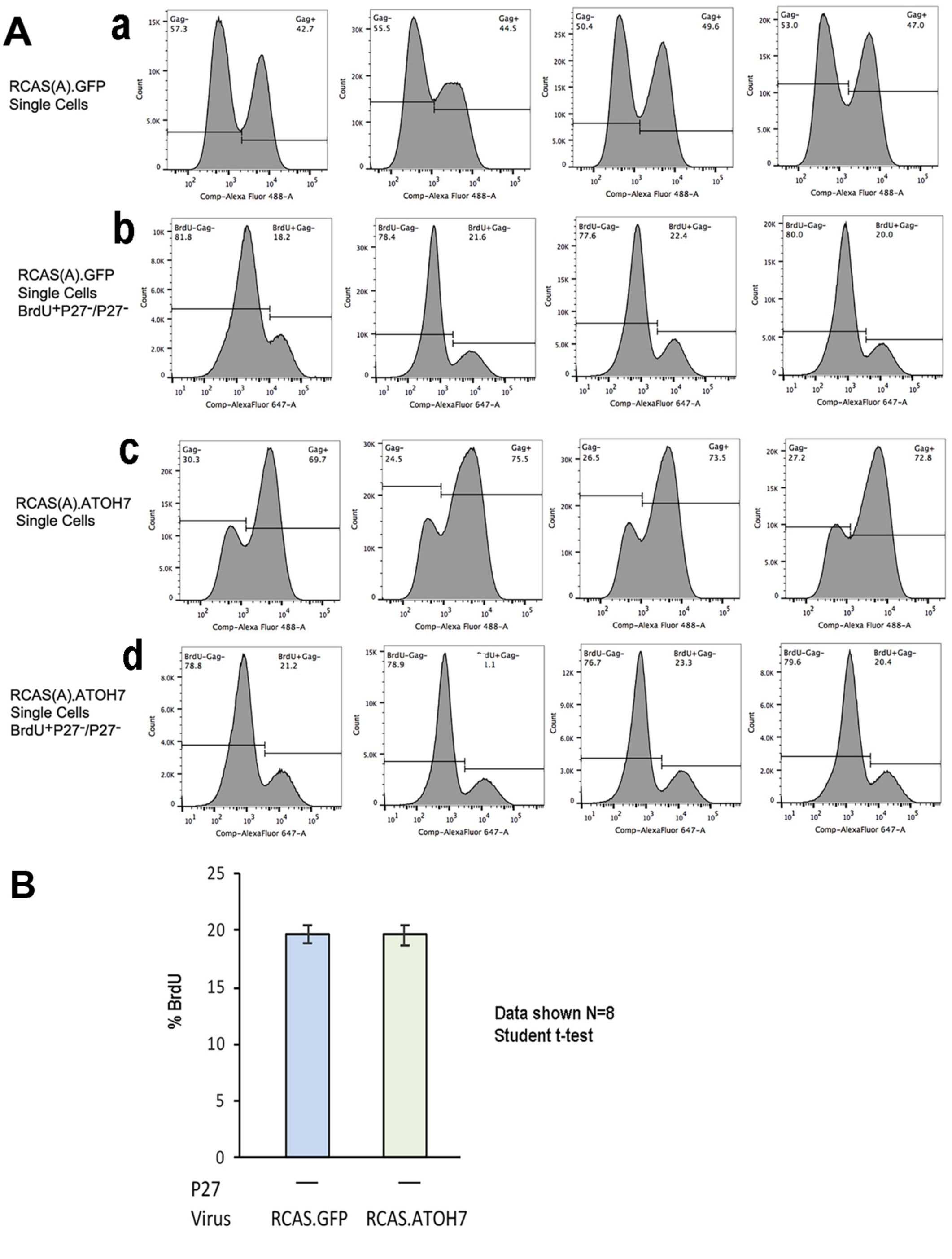

Sup. Figure 5. Quantification of viral infection impact on retinal cell proliferation.

(A) Representative flow cytometry profiles of stage 35 retinas infected with the control virus and ATOH7 virus at stage 17. (a, c) show gating of anti-gag antibody P27-postive and P27-negative cells. (b, d) show subsequent gating of BrdU labeling among P27-negative non-infected cells from control virus (b) or ATOH7 virus (d) infected retinas. (B) Quantification of BrdU incorporation among P27-negative non-infected cells in retinas infected by the control and ATOH7 viruses. Independent samples analyzed, N=8 for both. No statistical difference was detected, p=0.992 by Student T-test.

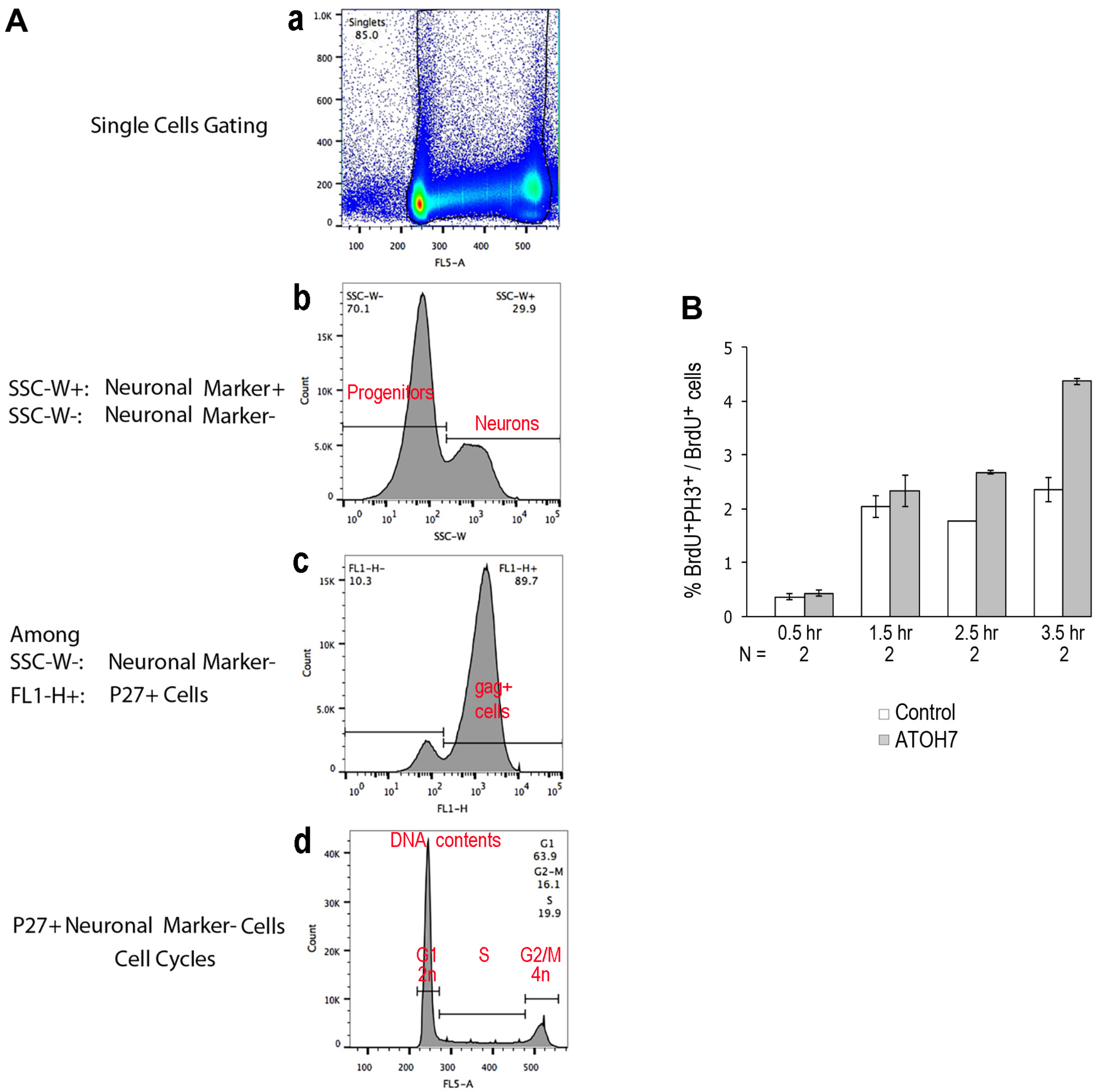

Sup. Figure 6. Flow cytometry analyses of cell cycle progression.

(A) Step-wise flow cytometry analyses of cell markers and DNA contents. An example is presented for the experiment shown in Figure 7A. Step (a) shows gating of single cell population. Step (b) shows labeling profiles for combined neuronal markers, including Islet-1, NF68, Visinin, Brn3a, and AP2α. Only neuronal marker-negative cells, "progenitors", were used for the next step. Step (c) selects viral infected gag-positive (P27+) progenitor cells for further analyzed. Step (d) shows DNA content analysis of gag-positive progenitors and their cell cycle distribution.
(B) Effect of elevating ATOH7 on progression from S phase to M phase of the cell cycle. Retinas were infected with viruses at stage 10 and pulse labeled with BrdU at stage 30. Bar graph shows BrdU and PH3 double positive cells, i.e. M phase cells, among BrdU-labeled cohort cells at different chase intervals after the BrdU pulse. Results of duplicate samples (N=2) are shown.
